# Adipose Mesenchymal Stem Cells Isolated after Manual or Water-jet-Assisted Liposuction Display Similar Properties

**DOI:** 10.3389/fimmu.2015.00655

**Published:** 2016-01-18

**Authors:** Claire Bony, Mailys Cren, Sophie Domergue, Karine Toupet, Christian Jorgensen, Danièle Noël

**Affiliations:** ^1^U1183, INSERM, Hôpital Saint-Eloi, Montpellier, France; ^2^UFR de Médecine, Montpellier University, Montpellier, France; ^3^Chirurgie Plastique Reconstructrice et Esthétique, Hôpital Gui de Chauliac, Montpellier, France; ^4^Service d’Immuno-Rhumatologie Thérapeutique, Hôpital Lapeyronie, Montpellier, France

**Keywords:** mesenchymal stem cells, adipose tissue, liposuction, DTH model, immunosuppression

## Abstract

Mesenchymal stem or stromal cells (MSC) are under investigation in many clinical trials for their therapeutic potential in a variety of diseases, including autoimmune and inflammatory disorders. One of the main sources of MSCs is the adipose tissue, which is mainly obtained by manual liposuction using a cannula linked to a syringe. However, in the past years, a number of devices for fat liposuction intended for clinical use have been commercialized but few papers have compared these procedures in terms of stromal vascular fraction (SVF) or adipose mesenchymal stromal cells (ASC). The objective of the present study was to compare and qualify for clinical use the ASC obtained from fat isolated with the manual or the Bodyjet^®^ water-jet-assisted procedure. Although the initial number of cells obtained after collagenase digestion was higher with the manual procedure, the percentage of dead cells, the number of colony forming unit-fibroblast and the phenotype of cells were identical in the SVF at isolation (day 0) and in the ASC populations at day 14. We also showed that the osteogenic and adipogenic differentiation potentials of ASCs were identical between preparations while a slight but significant higher *in vitro* immunosuppressive effect was observed with ASCs isolated from fat removed with a cannula. The difference in the immunomodulatory effect between ASC populations was, however, not observed *in vivo* using the delayed-type hypersensitivity (DTH) model. Our data, therefore, indicate that the procedure for fat liposuction does not impact the characteristics or the therapeutic function of ASCs.

## Introduction

Mesenchymal stem or stromal cells (MSC) are multipotent progenitor cells that are defined by their capacity to differentiate into chondrocytes, osteoblasts, adipocytes, and the expression of several markers, namely CD73, CD90, CD105 and lack of CD11b/CD14, CD19, CD34, CD45, and HLA-DR. They are also characterized by their trophic activity through the secretion of various factors, such as growth factors, chemokines, or cytokines. Thanks to these particular properties, they have been shown to exert a variety of therapeutic effects. They display proliferative, anti-fibrotic, pro-angiogenic, anti-apoptotic, anti-bacterial, and immunosuppressive functions that have been largely described *in vitro* and *in vivo*. After injection, the therapeutic effect of MSCs has been reported in many pre-clinical models of various diseases, notably in degenerative, autoimmune, or inflammatory diseases (1). Even though MSCs may be isolated from various tissue sources, most of these studies investigated the effect of MSCs that were isolated from bone marrow, umbilical cord, or fat. Adipose tissue-derived MSCs, also called adipose stromal cells (ASCs), share the characteristics of MSCs from other tissues, except for the expression of some markers: they express CD34 in the native tissue but not CD106 (2, 3). Because of the accessibility and the amount of fat tissue that can be recovered as well as the high yield of stem cells within the tissue, ASCs are evaluated in many clinical trials.

Adipose stromal cells are obtained after seeding the stromal vascular fraction (SVF) and expansion in culture for several days. The SVF corresponds to a heterogeneous population of cells generated after enzymatic digestion of the adipose tissue and removal of the mature adipocytes. Typically, the adipose tissue is harvested after lipoabdominoplasties or liposuctions. The two procedures, surgical resection or liposuction, are equivalent with regard to the ASC yield but liposuction may be superior for some tissue-engineering purposes, particularly because of the higher percentage of viable cells (4). The standard liposuction procedure currently used in clinics consists of using a cannula with a 10 ml syringe as described by Coleman (5). This procedure used for fat grafting is safe and routinely used in surgery for the adjuvant treatment of burn wounds, venous ulcers, diabetic ulcers, and burn scars. However, in the past years, numerous devices have been developed with the aim of standardizing the sampling, increasing the yield of progenitor cells, and improving the safety of fat removal. Specifically, the water-jet-assisted liposuction procedure is reported to provide a favorable esthetic outcome combined with high tissue protection and lower risk of cardiovascular side effects due to lowered amount of infiltrated fluid compared with conventional liposuction procedures (6). The water-jet-assisted liposuction Body-jet^®^ eco device is based on a liposuction tube connected to a negative-pressure pump and a water pump, which can inject a fan-shaped water jet during liposuction. This harvesting method has been reported to gently detach adipocytes from the fat tissue while reducing tissue damage and increasing cell viability and progenitor cell yield (7). Few studies compared the effectiveness of the different technologies on the amounts of cells recovered after liposuction (SVF or ASCs), expansion rates, or functional characteristics of the cells (8, 9).

The process for ASC isolation from subcutaneous abdominal fat, expansion, qualification has been validated by regulatory agencies for medicines and health products (10). However, no information is available on the comparison of different procedures used for fat withdrawal on the quality and functionality of ASCs cultured under good-manufacturing-practice (GMP) conditions. The aim of this study was, therefore, to compare two techniques for fat harvesting: the manual liposuction and the water-jet-assisted liposuction Bodyjet^®^ device on several parameters allowing qualification of ASCs for their use in clinical trials.

## Materials and Methods

### Ethics Statement

Adipose tissue was obtained from healthy patients undergoing abdominal liposuction after written informed consent signed by the patients as approved by the French Ministry of Research and Innovation and the Personal data Protection ethics Committee (CPP) of Languedoc-Roussillon (approval AC-2010-1200).

Animal experimentation was conducted in agreement with the recommendations of Languedoc-Roussillon Regional Ethics Committee on Animal Experimentation. Approval of the protocol was given by the Languedoc-Roussillon Regional Ethics Committee.

### Harvesting of Fat Tissue

Eight women were included in the study and the two techniques were used for each patient. The lipoaspirates were collected on both side of the umbilic in each patient. Liposuction of subcutaneous abdominal fat was carried out in patients under general anesthesia by an experienced surgeon.

#### Manual Liposuction

Manual liposuction was performed using a 3 mm cannula and 10 mL syringe with low aspiration (−290 mm Hg) as already described (5). The lipoaspirates were collected into two syringes per patient.

#### Water-jet-Assisted Liposuction

The water-jet-assisted liposuction procedure was carried out following supplier’s recommendations (Body-jet^®^ eco, Human Med AG, Schwerin, Germany) (6). Briefly, after a small incision in the abdominal wall, the Biofill cannula was introduced into the adipose tissue. The cannulas for harvesting had a diameter of 3.8 mm. An infiltration flow of saline containing epinephrine was applied to help gently detach the fat cells from the tissue, with simultaneous suction (−375 mmHg). The fat tissue was aspirated in a collector device and then transferred into a 50 mL syringe, which was aseptically closed. Whatever the procedure, the time from tissue harvest to SVF isolation was <2 h.

### Cell Isolation and Expansion

The recovered fat tissues were transferred to 50 mL Falcon tubes and weighted. For enzymatical dissociation, an equal volume of 250 U/mL of collagenase type II (Ref C6885, Sigma, Saint-Quentin Fallavier, France) was added to the fat tissue and incubated at 37°C for 45 min under agitation as previously described (11). Cell suspensions were filtered using a sterile tea strainer and centrifuged at 300 × *g* for 10 min. The SVFs were collected, treated with erythrocyte lysis buffer (155 mmol/L NH_4_Cl, 10 mmol/L KHCO_3_, 0.11 mmol/L EDTA) and filtered successively through 100, 70, and 40 μm porous filters (Cell Strainer, BD Biosciences, le Pont-de-Claix, France). Cells were centrifuged at 300 × *g* for 5 min and resuspended in expansion medium (α-MEM medium supplemented with 100 U/mL penicillin/streptomycin, 2 mmol/mL glutamine, 10% fetal calf serum and 1 ng/mL bFGF) (R&D Systems, Abingdon, UK). Quantification of cells was performed using a hemocytometer and cells were plated at 4000 cells/cm^2^ for 7 days. On day 7, cells were trypsinized, counted, and replated in expansion medium at the density of 2000 cells/cm^2^ for another period of 7 days (end of passage 1).

### Cell Culture Characteristics

Colony forming unit-fibroblast (CFU-F) efficiency was determined by seeding freshly isolated cells or ASCs at passage 1 at a concentration of 15 and 75 cells/cm^2^ in 100-mm plates for 14 days. Cell colonies were fixed with methanol for 10 min, dried and stained with Giemsa solution for 45 min, rinsed with distilled H_2_O, and let to dry. Colonies of more than 50 cells were counted.

Proliferation was measured as number of cell doubling per passage using the formulae: CD = ln(Nf/Ni)/ln2 where Nf is the final number of cells and Ni the initial number of cells. Population doubling time was calculated using the formulae: PDT = CT/CD; CT being the culture time.

### Phenotypic Characterization

Freshly isolated cells from the SVF and ASCs recovered at passage 1 (1 × 10^6^ cells) were suspended in phosphate buffered saline (PBS) containing 0.2% bovine serum albumin (BSA). Cells were then incubated with conjugated antibodies specific for CD11b, CD14, CD31, CD34, CD44, CD45, CD73, CD90, CD105, and HLA-DR and isotype-matched control antibodies (BD Biosciences) for 30 min on ice. Apoptotic cells were detected by FITC-conjugated Annexin V and 7AAD (BD Biosciences). Briefly, cells were washed twice with cold PBS, then suspended in 1X Annexin V binding buffer at 1 × 10^6^ cells/mL and incubated with FITC-Annexin V and 7AAD for 15 min at room temperature. Labeled cell acquisition was performed on a LSR Fortessa flow cytometer and data were analyzed using FACSDIVA 6.0 software (Becton Dickinson, San Jose, CA, USA).

### Adipogenic and Osteogenic Differentiation

For adipogenic differentiation, ASCs at passage 1 were plated at 8 × 10^3^ cells/cm^2^ in expansion medium. At confluence (between days 3 and 5), the medium is replaced by the expansion medium (negative control) or the differentiation medium consisting in DMEM:F12 supplemented with 5% FCS, 100 U/mL penicillin (Lonza, Verviers, Belgium), 100 μg/mL streptomycin (Lonza), 16 μM biotin, 18 μM pantothenic acid, 100 μM ascorbic acid, 60 μM indomethacin, 450 μM IBMX, 1 μM dexamethasone, and 10^−6^ M rosiglitazone (all from Sigma). The medium was changed every 3 days for 21 days (from cell seeding). Differentiation was assessed by visualization of lipid droplets. Cells were fixed with 3% glutaraldehyde for 1 h, rinsed twice with H_2_O and 60% isopropanol and then stained with Oil Red O dye (Sigma) for 2 h. Staining was then extracted by incubation with isopropanol for 10 min and quantified with a spectrophotometer at 540 nm.

For osteoblastic differentiation, ASCs at passage 1 were plated at 3 × 10^3^ cells/cm^2^ in expansion medium. At confluence (between days 5 and 7), the expansion medium (negative control) or the differentiation medium was added. Osteogenic medium consists in DMEM containing 10% FCS, 2 mM glutamine (Lonza), 100 U/mL penicillin (Lonza), 100 μg/mL streptomycin (Lonza), 50 μg/mL ascorbic acid, 100 nM dexamethasone, and 3 mM NaH_2_PO_4_ (all from Sigma). The medium was changed every 3 days for 21 days (from cell seeding). Osteogenesis was assessed by visualization of the mineralized matrix after staining with 2% Alizarin Red S solution. Cells were rinsed with PBS, fixed with 95% ethanol for 30 min and incubated with Alizarin Red S solution for 5 min. Quantification of hydroxyapatite deposition was performed by incubation with 10% cetylpyridinium chloride for 10 min and the optical density (OD) was measured by a spectrophotometer at 562 nm.

### Immunosuppressive Assay

Adipose stromal cells in proliferative medium were seeded in 96 wells plates at two densities (2 × 10^4^ or 4 × 10^4^ cells). Proliferative medium was IMDM supplemented with 2 mM l-glutamine, 100 U/mL penicillin, 100 μg/mL streptomycin, 0.1 mM non-essential amino acids, 0.25 mM β-2-mercaptoethanol, 1 mM sodium pyruvate, 10% inactivated FCS, and 20 mM Hepes. Peripheral blood mononuclear cells (PBMC) were isolated using Ficoll gradient from blood samples. PBMC were added in the wells containing the ASCs at the concentration of 2 × 10^5^cells/well and were activated with 2.5 μg/mL phytohemagglutinin. Cell cultures were incubated for 72 h. Proliferation of T lymphocytes was measured using the Cell Proliferation ELISA BrdU chemiluminescent kit (Roche Diagnostics, Meylan, France). Results are expressed as the percentage of proliferation of stimulated T cells and normalized to 100% for activated T cells.

### Delayed-Type Hypersensitivity Response

BALB/c mice of 7 weeks old were immunized by injection of 100 μg chick ovalbumin (cOVA) emulsified in 100 μL complete Freund’s adjuvant at the base of the tail. After 5 days, delayed-type hypersensitivity (DTH) response was tested by challenging with 30 μg cOVA in 30 μL physiological saline solution in the right footpad. ASCs (2.5 × 10^5^ cells) were mixed with the cOVA solution before injection. The left footpad was injected with 30 μL saline solution as a negative control. At day 6, the thickness of the footpads was measured using a caliper and thickness increment was calculated as the difference between the immunized footpads at day 6 (right-left) and the unimmunized footpads at day 0 (right-left).

### Statistics

Statistical analysis was performed with GraphPad Software (San Diego, CA, USA). Comparison between groups used Shapiro–Wilk normality test. Data were compared using the Mann–Whitney’s test for non-parametric values. Results were expressed as the mean ± SEM (standard error of the mean); a *p*-value ≤0.05 was considered significant.

## Results

### Isolation of ASCs from Lipoaspirates Obtained by Manual or Warterjet-Assisted Techniques

**Figure 1:**
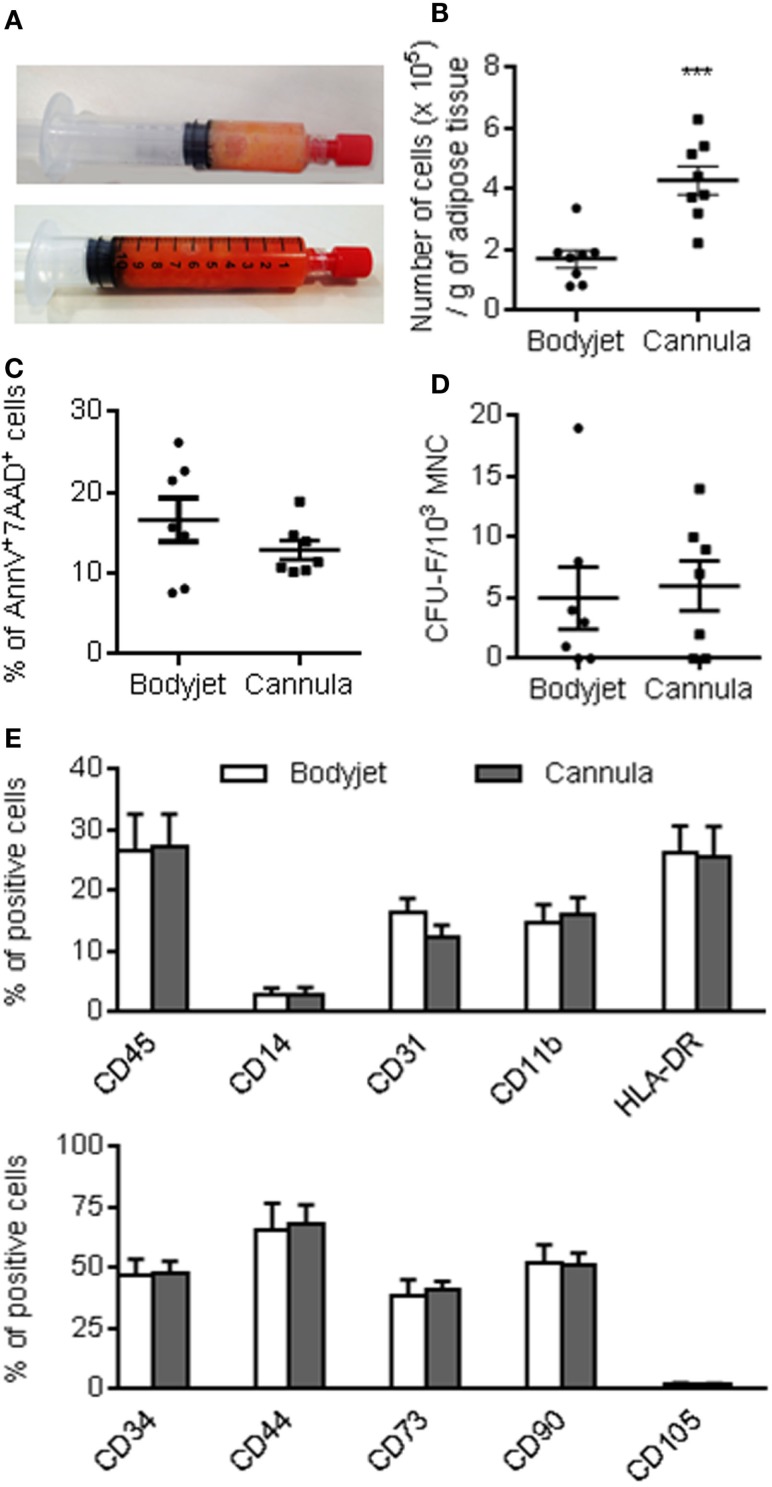
**Characteristics of the stromal vascular fraction at isolation**. **(A)** Representative photographs of fat after water-jet-assisted (Bodyjet) or manual (cannula) lipoaspiration (upper and lower panels, respectively). **(B)** Average number of mononuclear cells (MNC) obtained after collagenase digestion of the fat tissue removed by the two procedures. **(C)** Percentage of dead cells in the stromal vascular fraction (SVF) as evaluated as annexinV^+^ and 7AAD^+^ cells. **(D)** Average number of colony forming unit-fibroblast (CFU-F) in the MNC fraction of the SVF. **(E)** Immunophenotype of the SVF analyzed by flow cytometry, as expressed as percentage of viable cells positive for the indicated markers. *n* = 7–8 biological replicates; ***p < 0.001.

With the aim to compare the characteristics of ASCs isolated from fat tissue obtained after cannula- or water-jet-assisted procedures, we performed the two lipoaspiration procedures on the same patients. Eight women were included in the study and lipoaspirates from abdominal subcutaneous fat were withdrawn. The fat tissue obtained after water-jet-assisted lipoaspiration was generally less reddish than that obtained after the manual technique (Figure 1A). This was likely due to the high quantity of saline that was injected into the adipose tissue and discarded before fat tissue collection. After enzymatic dissociation of fat and SVF recovery, the cell yield was significantly different: 1.71 ± 0.81 and 4.29 ± 0.46 × 10^5^ cells/g tissue with the Bodyjet and manual procedure, respectively (Figure 1B). Viability of the cells was similar between the two procedures: 16.64 ± 2.72 and 12.9 ± 1.2% of AnnexinV^+^7AAD^+^ dead cells for the water-jet- or cannula-assisted technique, respectively (Figure 1C). We also determined the number of stem cells in the SVF suspensions by evaluating the number of CFU-F. The percentage of CFU-F in the SVF was identical in the two types of samples and estimated at 0.5 ± 0.2.5 and 0.6 ± 0.2% in the mononuclear cell fraction after Bodyjet and manual procedure, respectively (Figure 1D). Finally, we evaluated by fluorescence-activated cell sorting analysis whether the lipoaspiration procedures modified the phenotype of the SVF. Identical percentages of cell subtypes were detected in the two types of preparations, which contained 12–17% of CD31^+^ endothelial cells, 27% of CD45^+^ hematopoietic cells and 47% of ASC-containing CD34^+^ cells (Figure 1E). Other markers of hematopoietic subpopulations identified the presence of 2.5% of CD14^+^ monocytes, 15% of CD11b^+^ monocytes/macrophages, and 25% of HLA-DR^+^ cells. Evaluation of the markers of ASCs revealed the proportion of 68% of CD44^+^ cells, 40% of CD73^+^ cells, 50% of CD90^+^ cells, and 1.5% of CD105^+^ cells. More than 98% of the CD44^+^CD73^+^CD90^+^ cells were CD34^+^ confirming that ASCs were in the CD34^+^ cell population.

### Characteristics of ASCs After Expansion

**Figure 2:**
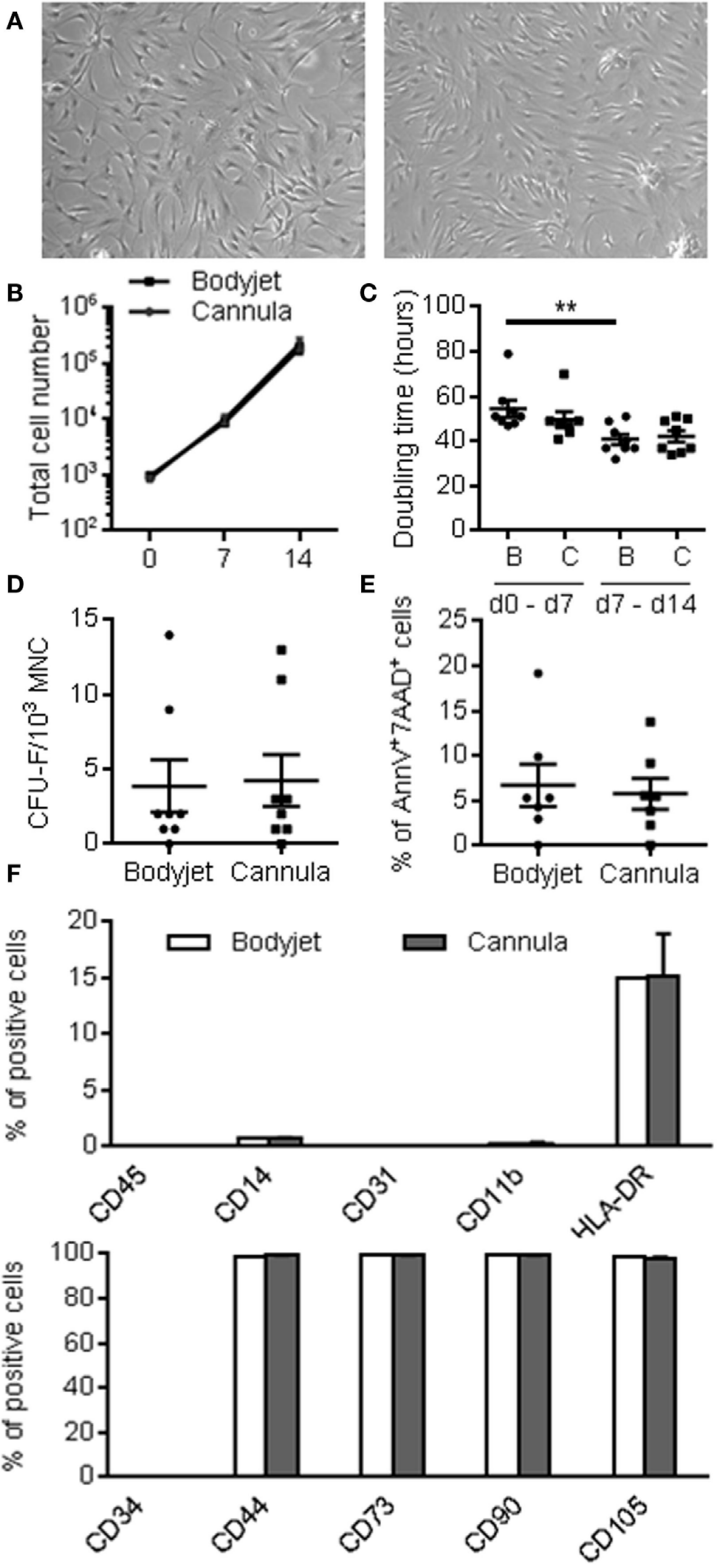
**Characteristics of ASCs after 14 days of expansion**. **(A)** Representative photographs of ASCs isolated from fat obtained after water-jet-assisted (Bodyjet; B) or manual (cannula; C) lipoaspiration (left and right panels, respectively). **(B)** Cumulative number of ASCs obtained from fat removed by the two procedures during 14 days culture. **(C)** Average doubling time during the first (days 0–7) or second (days 7–14) week of culture. **(D)** Average number of colony forming unit-fibroblast (CFU-F) in the ASC populations. **(E)** Percentage of dead cells in the ASC populations as evaluated as annexinV^+^ and 7AAD^+^ cells. **(F)** Immunophenotype of the ASC populations analyzed by flow cytometry, as expressed as percentage of viable cells positive for the indicated markers. *n* = 7–8 biological replicates; **: *p* < 0.01.

Stromal vascular fraction obtained after manual or water-jet-assisted procedures were plated at the density of 4000 cells/cm^2^ and cultured to confluency for 7 days. After trypsination, reseeding and culture for 7 additional days, the adherent cells from the two types of techniques displayed similar elongated morphology with fibroblastic-like appearance (Figure 2A). Growth kinetics were identical between the two cell preparations. The cell number was multiplied by a 230-fold factor in 2 weeks (Figure 2B). Doubling time evaluation indicated similar values for water-jet-assisted or manual procedures: 54.5 ± 10.5 and 47.7 ± 9.4 h between days 0 and 7, respectively, and 40.9 ± 6.5 and 42.2 ± 7.2 h between days 7 and 14 (Figure 2C). The doubling time of ASCs isolated with the Bodyjet technique was significantly reduced between day 7 and day 14. We also determined the number of CFU-F in the adherent cell population at day 14. The same percentage of CFU-F was detected after expansion of cells isolated by the two procedures: 4% of the population was able to form colonies (Figure 2D). The viability of the cells after expansion was very good with 6% of dead cells and again no significant differences between the two techniques (Figure 2E). Finally, we analyzed the phenotype of cells after 14 days of culture. We noticed absence of endothelial and hematopoietic cells, as revealed by the lack of expression of specific markers for CD31, CD34, CD45, CD11b, and less than 1% CD14 (Figure 2F). By contrast, more than 99% of the cells expressed CD73, CD90, CD105, and CD44, while 15% of the cells expressed HLA-DR. Importantly, more than 99% of the CD44^+^, CD73^+^, CD90^+^, and CD105^+^ cells were in the CD34^−^ cell population (data not shown). Altogether, these results indicate that similar yield of ASCs was obtained upon culture, whatever was the procedure for fat isolation.

### Differentiation Potential of ASCs

**Figure 3:**
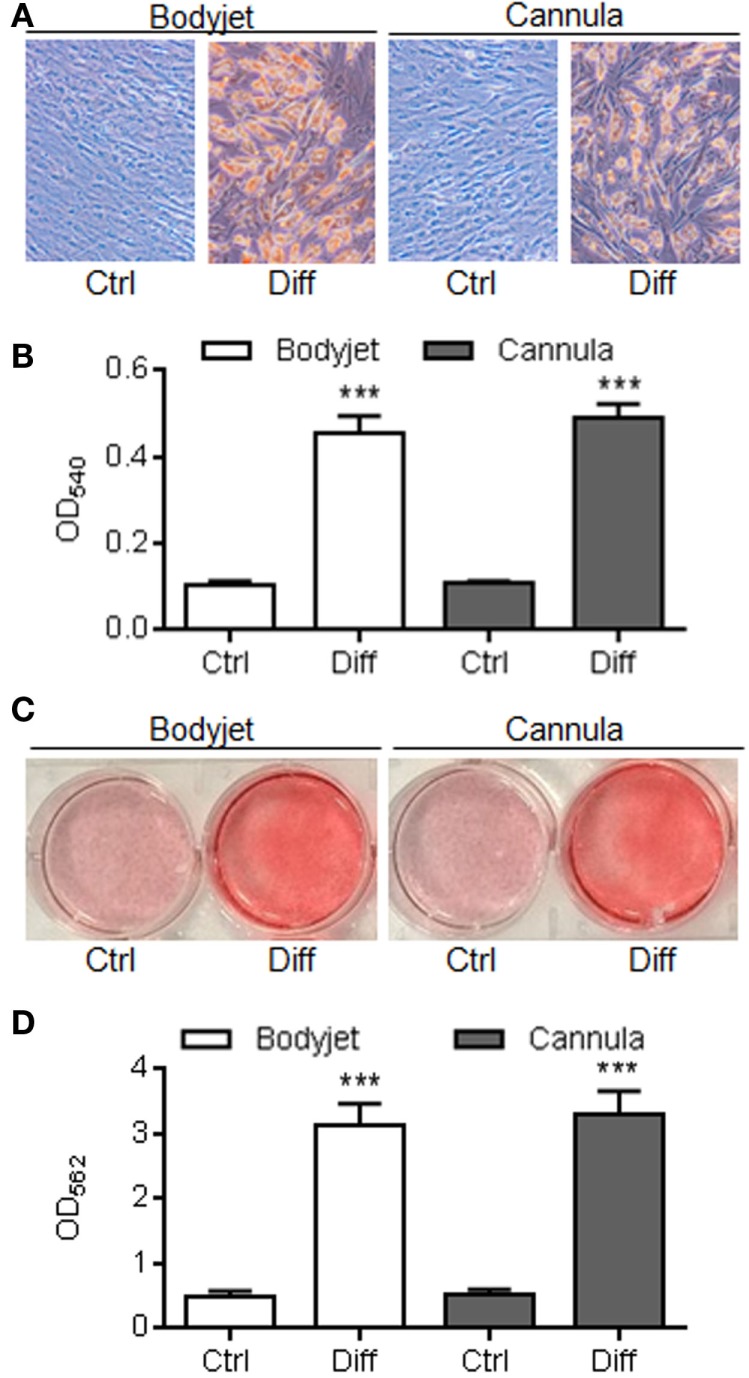
**Differentiation potential of ASCs**. **(A)** Representative photographs of ASCs isolated from fat obtained after water-jet-assisted (Bodyjet) or manual (cannula) liposuction. Cells were cultured in expansion medium (Ctrl) or adipogenesis inductive conditions (Diff) and stained with Oil Red O. **(B)** Quantification of Oil Red O dye after extraction from cultures in **(A)** and spectrometric analysis at the optical density (OD) of 540 nm. **(C)** Representative photographs of ASCs isolated from fat obtained by two techniques. Cells were cultured in expansion medium (Ctrl) or osteogenesis inductive conditions (Diff) and stained with Alizarin Red S. **(D)** Quantification of Alizarin Red S dye after extraction from cultures in **(C)** and spectrometric analysis at the optical density (OD) of 562 nm. *n* = 8 biological replicates; ***p < 0.001.

The differentiation potential of ASCs toward adipogenesis and osteoblastogenesis was investigated *in vitro* after 21 days of culture in inductive media. After adipogenesis induction, a high number of cells changed their morphology toward a rounder shape that accumulated lipid droplets (Figure 3A). In parallel, culture of the cells in expansion medium maintained the elongated fibroblastic morphology of the cells with no formation of lipid droplets. Lipid droplets were visualized after staining with Oil Red O. After extraction of the dye from the culture and spectrophotometric quantification, a highly significant increase of the OD measured at 540 nm was detected in the wells containing adipocytes as compared to the undifferentiated control (Figure 3B). The level of differentiation was, however, similar between ASC isolated from Bodyjet- or cannula-assisted techniques.

The potential to differentiate into osteoblasts was evaluated by culture in a medium containing a source of phosphate. Differentiation of ASCs and mineralization of the extracellular matrix were detected by Alizarin Red S staining. Whatever was the technique of fat isolation, ASC-derived osteoblasts highly mineralized the matrix as shown by red staining after culture in osteoinductive medium, while ASCs cultured in expansion medium were not able to differentiate (Figure 3C). Quantification of the dye after extraction nicely demonstrated a high staining in wells containing osteoblasts as compared to undifferentiated cells (Figure 3D). These data pointed out that the process for fat isolation did not impact the differentiation potential of ASCs.

### Immunosuppressive Potential of ASCs

**Figure 4:**
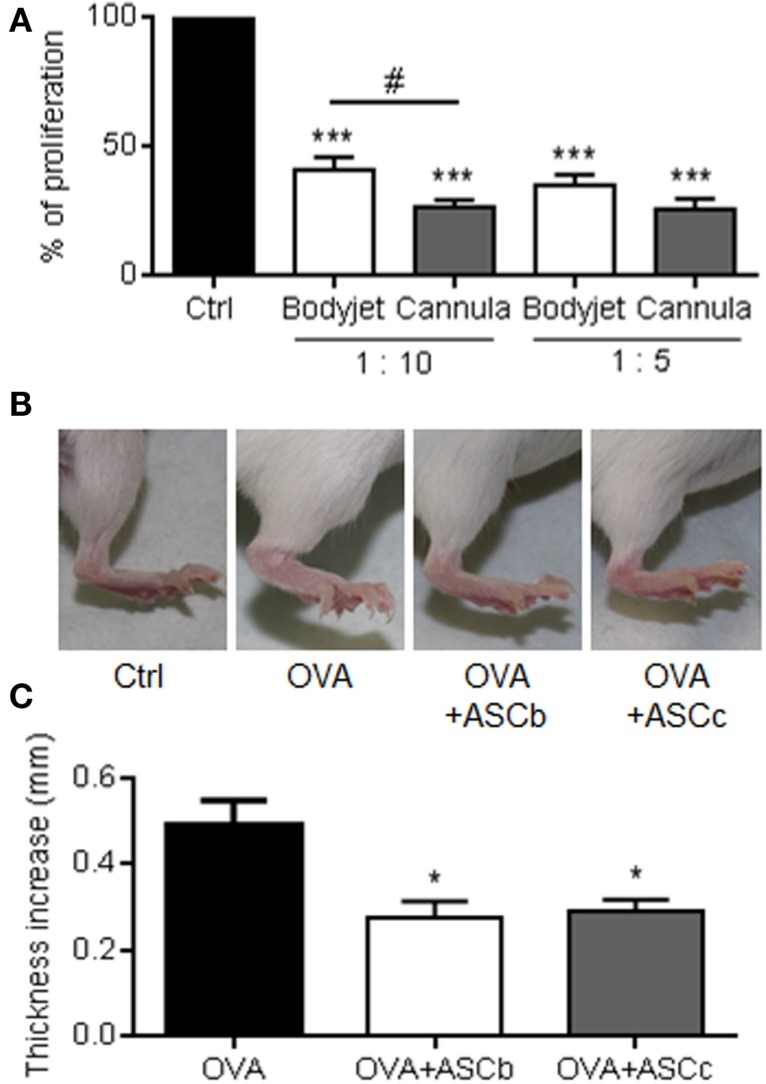
***In vitro* and *in vivo* immunosuppressive potential of ASCs**. **(A)** Immunosuppressive potential of ASCs isolated from fat obtained after water-jet-assisted (Bodyjet) or manual (cannula) liposuction. The proliferation of peripheral blood mononuclear cells (PBMC) cultured alone (Ctrl) or with ASCs at two ratio (1:10 and 1:5; ASC:PBMC) was quantified. *n* = 8 biological replicates. **(B)** Representative photographs of hind paws of mice immunized with PBS (Ctrl), ovalbumin (OVA) or OVA containing ASC isolated from fat obtained by Bodyjet (ASCb) or cannula (ASCc) lipoaspirations. **(C)** Average thickness of hind paws of mice immunized in **(B)**. *n* = 2 biological replicates in tetraplicates; *: *p* < 0.05; ***: *p* < 0.001;#: *p* < 0.05 Bodyjet versus cannula.

We then evaluated *in vitro* the immunosuppressive capacity of ASCs. After 72 h culture of ASCs with PHA-activated PBMCs at different ratios, we measured the proliferative level of PBMCs. As compared to the proliferation of activated PBMC cultured alone, addition of ASCs led to a significant decrease of the PBMC proliferation (Figure 4A). Although not significant, the highest 1:5 MSC:PBMC ratio decreased slightly the proliferation of PBMC. Interestingly, when comparing the level of proliferation of PBMCs after culture with ASCs obtained from the two procedures of fat isolation, a significant improvement of the immunosuppressive effect of ASCs was detected with ASCs isolated after cannula-assisted technique (Figure 4A).

We, therefore, evaluated *in vivo* the immunomodulatory potential of six ASCs isolated from two different fat samples recovered by the two procedures in the DTH model. In this model, the immunization of mice with ovalbumin (OVA) at the basis of the tail followed by a boost in the fat pad after 5 days induced a high inflammatory reaction 24 h later, as visualized by the increase of the foot pad thickness (Figure 4B). The injection of ASCs together with OVA at the time of the boost reduced the inflammatory reaction and the foot pad thickness. Thickness of the foot pads was measured with a caliper and the average measures confirmed that ASCs were able to significantly reduce the inflammatory response (Figure 4C). However, there was no significant difference between ASCs isolated from fat recovered using the water-jet-assisted or manual procedure. Indeed, although ASCs obtained after manual liposuction were slightly more immunosuppressive *in vitro*, our *in vivo* data indicated that both techniques for fat sampling allowed isolating ASCs that are efficient to significantly reduce the host immune response.

## Discussion

The interest of MSCs for regenerative medicine and the number of clinical trials based on the use of adipose-derived MSC are continuously growing. The processes for ASC isolation and expansion under GMPs have been optimized to ensure reproducibility, scalability, and safety of the cell cultures. Safety control and qualification of ASCs for batch release generally include viability tests, bacteriological controls, phenotypic analysis, and whenever possible functional analysis (clonogenicity, expression of specific factors) (12). However, impact of the techniques for fat liposuction on the amount of isolated ASCs, their phenotype and functions has been poorly investigated. Generally, adipose tissue liposuction is based on the Coleman’s method but the use of medical devices is being developed. Most of these clinical devices, which rely on different principles, have been intended for fat transfer in several indications, such as mammary reconstruction, defect lipofilling, or esthetic surgery. However, adipose tissue recovered with these devices may be used for SVF preparation and ASC isolation. A study has recently compared the Coleman’s standard lipotransfer technique with three commercially available devices, based on enzymatic dissociation (Cytori Celution from Cytori Ltd., UK and Lipokit Medikan from Medikan International Inc., Korea) or mechanic isolation (Fastem Corios from Corios Soc. Coop., Italy) of SVF cells (8). The authors demonstrated a significant superiority of the device-assisted lipoaspiration procedures in terms of frequency of SVF, number of clonogenic cells and multipotent ASC in the fat grafts; the enzymatic-based lipoaspiration procedures being superior to mechanical dissociation procedures. However, the study compared standard versus stem cell-enriched fat grafts but no direct comparison of SVF or ASCs isolated from the three different devices was performed. While the various procedures aimed at dissociating the fat tissue to recover the highest number of SVF cells, variability in results was observed. In the present study, we compared SVF or ASCs expanded from fat isolated with the manual liposuction or the Bodyjet^®^-assisted procedures, which is another technique for fat removal.

We demonstrated that the characteristics of cells recovered by the two procedures are similar. Whatever was the procedure for fat isolation, the SVF preparations displayed equivalent number of viable cells, CFU-F yield, and immunophenotype. All these parameters were in the range of those obtained with previous work and other medical devices commercially available (8, 13). The total number of cells per gram of fat, which was recovered after enzymatic digestion, was, however, higher using the manual procedure. We have to underline that due to the large amounts of fat collected, only part of the tissue was enzymatically dissociated and this is why cell numbers were normalized to the weight of tissues processed. The quantity of fat recovered with the water-jet-assisted technique was usually approximately two to three times more abundant than that obtained with the manual procedure. This was likely due to the infiltration of fat with the saline solution during the procedure. Indeed, if we take into account the larger quantity of recovered fat, the total number of cells is equivalent between the two techniques. This is further indicated by the similar quantity of CFU-F progenitor cells in the two cell preparations. After ASC expansion and characterization at the end of passage 1, we observed similar proliferative rates, population doubling times, numbers of CFU-F, cell viability, and immunophenotypes between the two procedures. We here confirmed that the native ASC population was in the CD34^+^ cell fraction in the SVF while this marker, which is negatively correlated with proliferation, is lost upon culture (2). Although some studies reported similar observations by analyzing independently water-jet-assisted or mechanical liposuction procedures (6, 14) or comparing medical devices among themselves (8, 13), the present study is the most exhaustive one. The reported parameters are usually evaluated for batch release in GMP production of advanced cell therapy medicinal products. Furthermore, the Bodyjet^®^-assisted liposuction was compared to the commonly used gentle Coleman’s method, and results clearly indicate that ASC yield is not dependent on the technique used for fat isolation.

Regarding the functional properties of ASCs, we first characterized their potential to differentiate toward osteoblasts and adipocytes since ASCs are being evaluated for repair of bone defects and lipodystrophies in several clinical trials (see clinicaltrials.gov). We report equivalent potential for ASCs isolated with the two procedures confirming that expanded ASCs were functionally effective. Chondrogenic potential was not investigated because ASCs display a lower capacity to differentiate toward chondrocytes (3, 15). This lower chondrogenic capacity has been attributed to the absence of expression for TGF-βRI (16). Our results are in concordance with previous studies reporting the preservation of the adipogenic and osteogenic potential of ASCs isolated from fat obtained using different methods of liposuction (8, 14). Finally, we evaluated the immunosuppressive capacity of ASCs as possible cell sources for the treatment of diseases of the immune system. To our knowledge, the immunosuppressive potential of ASCs has not been previously investigated in studies comparing the efficacy of fat liposuction procedures. Interestingly, ASCs isolated from manual liposuctions displayed a slight but significantly higher immunomodulatory potential than those from Bodyjet^®^-assisted liposuctions. The reason for these discrepancies is not understood. It cannot be attributed to the environment since abdominal fat samples were withdrawn from the same subjects. However, the differences were leveled out in the DTH model, suggesting that *in vivo* licensing of ASCs plays an important role for their functionality. This is further supported by a recent study reporting that heterogeneity of the immunomodulatory activity of MSCs at the clonal level may be erased by licensing the cells (17). Upon pretreatment of MSCs with IFN-γ and TNF-α, an increase of their immunosuppressive function was observed both *in vitro* and *in vivo*. Pre-activation of MSCs resulted in enhanced levels of *Nos2* gene and NO inactivation by a specific inhibitor completely reversed the immunosuppressive effect of MSCs. Indeed, although liposuction procedures may slightly impact the immunosuppressive potential of ASCs, ASC licensing by the *in vivo* inflammatory environment allows to get rid of individual variability.

The present study indicates that gentle water-jet-assisted liposuction is similar to the commonly used Coleman’s method in terms of SVF and ASC recovery. Compared to direct fat excision method, liposuction performed using the Coleman’s method yield less ASCs, while power-assisted liposuction has been shown to yield same numbers of ASCs with similar characteristics (18, 19). Another study has reported that contrary to fat resection, manual liposuction preserves the microvascular network (20). Here, we present evidence that water-jet-assisted liposuction yield less total cells/g of fat tissue than the Coleman’s procedure but the number of CFU-F, which represents the stem cell fraction of the tissue, is equivalent. Moreover, it was previously reported that Bodyjet-assisted liposuction was as safe as the traditional liposuction technique with no hypovolemia, hemorrhages, or infections (21). The technique reduces pain and ecchymosis, indicating fewer traumas to tissue nerves and blood vessels than manual liposuction. The advantages of the water-jet-assisted procedure over manual liposuction are the rapidity of fat tissue collection, the safety, and the standardization of the process. For ASC isolation in clinical production centers, where standardized procedures are required, the possibility to optimize and standardize the fat sampling technique, minimizing variations due to the clinician, is of upmost importance.

## Author Contributions

Design of the study: DN, CJ. Acquisition of data: CB, MC, SD, KT. Data analysis: CB, MC, KT, DN. Manuscript preparation: DN. All authors reviewed the manuscript and gave final approval for the work.

## Conflict of Interest Statement

The authors declare that the research was conducted in the absence of any commercial or financial relationships that could be construed as a potential conflict of interest.
